# Glioblastoma Subclasses Can Be Defined by Activity among Signal Transduction Pathways and Associated Genomic Alterations

**DOI:** 10.1371/journal.pone.0007752

**Published:** 2009-11-13

**Authors:** Cameron Brennan, Hiroyuki Momota, Dolores Hambardzumyan, Tatsuya Ozawa, Adesh Tandon, Alicia Pedraza, Eric Holland

**Affiliations:** 1 Department of Neurosurgery, Memorial Sloan Kettering Cancer Center, New York, New York, United States of America; 2 Brain Tumor Center, Memorial Sloan Kettering Cancer Center, New York, New York, United States of America; 3 Department of Cancer Biology and Genetics, Memorial Sloan Kettering Cancer Center, New York, New York, United States of America; 4 Department of Neurology, Memorial Sloan Kettering Cancer Center, New York, New York, United States of America; 5 Department of Surgery, Memorial Sloan Kettering Cancer Center, New York, New York, United States of America; Baylor College of Medicine, United States of America

## Abstract

**Background:**

Glioblastoma multiforme (GBM) is an umbrella designation that includes a heterogeneous group of primary brain tumors. Several classification strategies of GBM have been reported, some by clinical course and others by resemblance to cell types either in the adult or during development. From a practical and therapeutic standpoint, classifying GBMs by signal transduction pathway activation and by mutation in pathway member genes may be particularly valuable for the development of targeted therapies.

**Methodology/Principal Findings:**

We performed targeted proteomic analysis of 27 surgical glioma samples to identify patterns of coordinate activation among glioma-relevant signal transduction pathways, then compared these results with integrated analysis of genomic and expression data of 243 GBM samples from The Cancer Genome Atlas (TCGA). In the pattern of signaling, three subclasses of GBM emerge which appear to be associated with predominance of EGFR activation, PDGFR activation, or loss of the RAS regulator NF1. The EGFR signaling class has prominent Notch pathway activation measured by elevated expression of Notch ligands, cleaved Notch receptor, and downstream target Hes1. The PDGF class showed high levels of PDGFB ligand and phosphorylation of PDGFRβ and NFKB. NF1-loss was associated with lower overall MAPK and PI3K activation and relative overexpression of the mesenchymal marker YKL40. These three signaling classes appear to correspond with distinct transcriptomal subclasses of primary GBM samples from TCGA for which copy number aberration and mutation of EGFR, PDGFRA, and NF1 are signature events.

**Conclusions/Significance:**

Proteomic analysis of GBM samples revealed three patterns of expression and activation of proteins in glioma-relevant signaling pathways. These three classes are comprised of roughly equal numbers showing either EGFR activation associated with amplification and mutation of the receptor, PDGF-pathway activation that is primarily ligand-driven, or loss of NF1 expression. The associated signaling activities correlating with these sentinel alterations provide insight into glioma biology and therapeutic strategies.

## Introduction

Glioblastoma (GBM) is the most common malignant brain tumor and is characterized by intratumoral heterogeneity, invasive growth pattern and poor response to treatment.[1]–[4] While GBM comprises approximately 25% of all brain tumors in adults, in absolute numbers it is still an uncommon cancer. This low absolute incidence combined with high morbidity, poor response rates and short survival times pose practical problems for clinical trial execution, particularly if therapy is anticipated to target a molecularly-defined subset of tumors. The current first-line treatment for GBM is radiation with alkylating chemotherapy (Temozolomide) given concurrently and then continued after radiation. This uniform first-line treatment approach contrasts with the wealth of molecular data on mutations, genomic aberrations and transcriptomal features in GBM which indicate potential therapeutic targets and resolve apparently distinct subclasses of these tumors.[3] We designed this investigation of signal transduction pathway activation in GBM with the expectation that tumor subclasses based on differential activation of glioma-relevant pathways would be of paramount utility for interpreting responses to therapies targeting these pathways, and potentially applicable for stratifying patients in clinical trials.

Historically, two subtypes of GBM were distinguished based on histologic grade at clinical presentation. Primary GBMs present initially as grade 4 tumors while secondary GBMs present as lower grade gliomas and progress to GBMs over time.[5] Although primary and secondary GBM differ in the frequency of molecular abnormalities seen, they draw largely from a common palette of events: amplification and activating mutations in EGFR, over-expression of PDGF and its receptors and loss of the tumor suppressors INK4a/ARF, p53 and PTEN are well-documented, recurrent mutations in these tumors.[1] Recent large-scale efforts to characterize the glioblastoma genome have identified additional recurrent alterations in genes not previously implicated in glioma, such as *ERBB2* and *IDH1* mutation in primary and secondary GBM respectively, and a significant incidence of mutation and genomic loss of *NF1*.[6]–[8] While there is hope that further sequencing will yield new therapeutic targets, it should be noted that single-agent therapy trials of inhibitors directed to the two most commonly altered receptors have not been successful in unselected populations.[9], [10] At least in some cases this may be due to failure of RTK inhibition to impact downstream or parallel signal transduction pathway activation.[11]

The importance of signal transduction activity downstream of tyrosine kinase receptors in glioma biology is emphasized by the fact that these pathways are abnormally active in these tumors and causal in their formation in mice.[12], [13] Therefore, there is significant interest in whether molecular subclasses of GBM might be identified based on distinct signaling characteristics, and whether such subclasses could be used to refine patient stratification in clinical trials or help interpret treatment responses. Several studies have subdivided GBMs by expression array analysis measuring total cellular mRNA levels.[14]–[19] However, because of differential translational efficiencies, total mRNA levels do not always correlate with protein levels. Moreover, most signaling activity is achieved by post-translational modifications of existing proteins such as Notch cleavage, phosphorylation of kinases, or stabilization of proteins such as beta catenin. To further complicate matters, signal transduction activity may affect, through feedback mechanisms, the translational efficiencies of mRNAs encoding proteins with effects on the oncogenic phenotype.[20], [21] Therefore, direct measurements of active signaling components at the protein level are critical to fully characterize the signal transduction state of the cell. Such measurements have been made on glioma samples and have shown correlation between the levels of active pathway components.[20], [22]

In this study, we have measured the relative protein levels of signaling molecules within pathways thought to be crucial to glioma biology among a panel of gliomas. This represents a more extensive proteomic analysis of signaling pathway members than has previously been done, and reveals three patterns of signaling pathway activation. Distinct patterns were each associated with EGFR and PDGF pathway activity while a third was associated with low levels of NF1 protein. We present an analysis of genomic features among tumors in each group, and show how these proteomically-defined subclasses of GBM compare with genomic subclasses arising from integrated analysis of primary GBM in data from The Cancer Genome Atlas.

## Results

### Specimens and protein analysis

27 glioma surgical samples were identified from a database of patients operated on at MSKCC and consented to the IRB-approved protocol. Clinical and pathologic characteristics are summarized in Table 1. Pathology included 20 GBM, 2 anaplastic astrocytomas, 4 oligodendrogliomas of which 2 were anaplastic tumors, and one tumor characterized as high grade glioma with glioneuronal elements. 11 GBM were recurrent after treatment (RT +/− temozolomide) and two of these were secondary GBM. The age range was from 32 to 74 (median 53).

**Table 1:** Patient and tumor characteristics.

ID	Age	M/F	Survival (weeks) from resection	Survival (weeks) from diagnosis	Pathology	Prior Treament
GBM.1	74	F	4	4	GBM	–
GBM.2	66	F	(6)	(35)	GBM	RT
GBM.3	71	F	12	34	GBM	RT
GBM.4	70	F	46	46	GBM	–
GBM.5	39	M	32	45	GBM	RT
GBM.6	67	F	150	150	GBM	–
GBM.7	42	M	138	138	GBM	RT
GBM.8	56	M	27	60	GBM	RT + temozolomide
GBM.9	40	M	14	206	Secondary GBM	PCV
GBM.10	52	M	35	35	GBM	–
GBM.11	71	M	32	32	GBM	–
GBM.12	53	F	5	44	GBM	RT + temozolomide
GBM.13	47	M	35	38	GBM, oligoastrocytoma features	–
GBM.14	59	M	21	47	GBM	RT + temozolomide
GNT.15	68	M	51	69	High grade glioneuronal tumor	RT
GBM.16	38	M	15	94	GBM	RT
GBM.17	60	F	49	49	GBM	–
GBM.18	32	F	(3)	(84)	GBM	RT, high dose thiotepa
GBM.19	49	M	85	85	GBM	–
GBM.20	71	M	212 +	212 +	GBM	–
AO.21	64	F	(218)	(218)	anaplastic oligodendroglioma	–
ODG.22	52	F	(6)	(475)	oligodendroglioma	–
AA.23	46	F	(76)	(544)	anaplastic astrocytoma	high-dose thiotepa
AO.24	45	M	194 +	198 +	anaplastic oligodendroglioma	–
ODG.25	42	F	153 +	153 +	oligodendroglioma	–
AA.26	47	F	35	254	anaplastic astrocytoma	RT
GBM.27	74	M	7	55	GBM	RT + temozolomide

Demographic and pathologic description for 27 study patients. Survival is shown from time of resection of study tumor: “+”  =  still alive and parentheses mark patients lost to follow-up. For recurrent tumors, prior treatment and survival from initial diagnosis.

Protein extracts from these tumors were analyzed by western blot for the activity of various signaling pathways related to glioma formation or stem cell character, totaling 55 antibodies for proteins in total and active forms (Table S1). Antibodies were selected based on known performance. Briefly, PDGF pathway activity was measured by PDGF ligand (PDGFB), PDGFRα, PDGFRβ and phospho-PDGFRβ. EGFR activity was assessed by antibodies specific for EGFR and phospho-EGFR. The downstream pathways interrogated included Ras, Akt, Notch, Wnt and SHH. The Akt pathway was interrogated by PTEN, total AKT, phospho-AKT and RHEB. Activation of mTOR was measured by phospho-S6 ribosomal protein (p-S6RP). The Ras pathway was interrogated by BRAF and total/phospho-MEK and ERK. Notch pathway activity was measured by the Notch ligands Delta (DLL1) and Jagged (JAG1), full length Notch receptors 1 and 2, cleaved Notch 1 and 2 and downstream Notch target HES1. The Wnt pathway was assessed by beta catenin levels and the SHH pathway by SHH levels. NF1 was assayed in preserved lysates at a later date, after mutations were reported for this gene in GBM.[7], [8] Quantified bands for 55 antibodies were normalized against actin or tubulin. HEB, Notch1 and Notch2 antibodies each generated two bands which were independently quantified. Therefore a total of 58 protein forms were quantified and normalized. Relevant western bands selected by size are summarized in Figure S1A, S1B, S1C, S1D, and quantified values normalized against actin are given in Table S2A, S2B, S2C.

### Principal Component Analysis and unsupervised clustering identify three patterns of signaling in GBM

**Figure 1:**
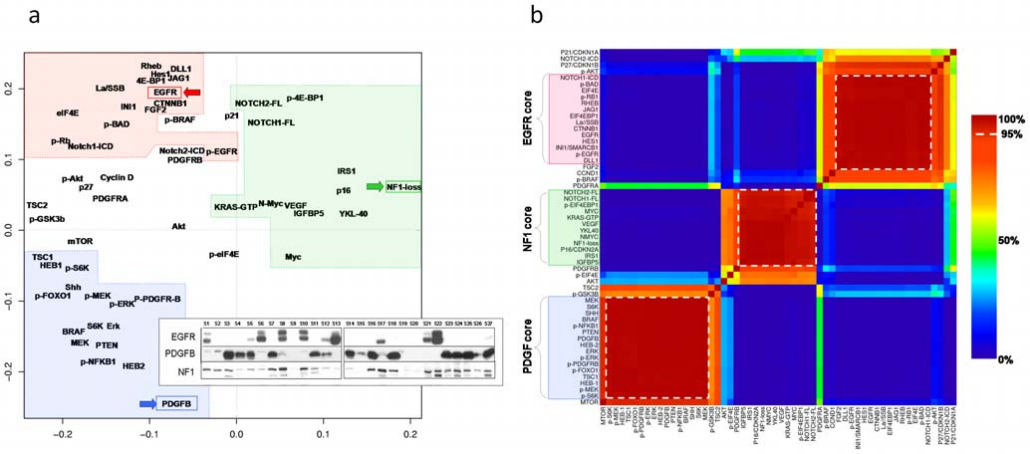
Analysis of quantified western data in 20 GBM identifies three signaling axes associated with EGFR overexpression, PDGFB overexpression, and loss of NF1. (A) Principal component analysis (PCA) of 56 proteins in 20 GBM samples by quantified western blot (see Methods). The first two components are plotted, accounting for 51% of variance in protein levels. PC2 strongly distinguishes proteins correlated with EGFR (red arrow) versus PDGFB (blue), while PC1 distinguishes a third pattern which is correlated with neither of these RTK pathways. Of note, NF1 appears to be silenced in this group (“NF1 loss”, zero minus standardized protein expression, green). Dashed lines bound proteins with significant co-expression by k-means clustering (see B). Inset shows the western bands confirming mutual exclusivity for EGFR expression, PDGFB expression and NF1 silencing. (B) K-means clustering of proteins confirms three statistically significant core clusters. Unsupervised k-means clustering of quantified protein levels in 20 GBM reveals 3 patterns of coordinate protein expression. The consensus matrix shown represents how often two proteins were co-clustered during 10,000 iterations, leaving out 15% of samples (n = 3) selected at random for each iteration. “Core” correlated proteins are those that show >95% co-clustering across iterations (dashed-lines). These define an EGFR group, a PDGFB group and a third non-EGFR/PDGF group which features NF1 loss. 3-way clustering was determined to be the best fit by consensus matrix stability and cophenetic correlation (see text, Figure S2).

As expected, overall activation of signal transduction pathways differed markedly between the glioma samples and the normal brain reference. While some pathways such as PI3K and MAPK were active nearly uniformly among gliomas, we sought to investigate whether relative differences might distinguish subclasses among the samples with GBM pathology. The overall pattern of protein expression and activation was first assessed by Principal Component Analysis (PCA). Quantified western data for 57 protein forms were standardized across the set of GBM samples for analysis (n = 20). We excluded p53 from analysis because common inactivating mutations can be associated with increased or decreased protein levels. For clarity in the figure, NF1 was represented as “NF1 loss” (zero minus standardized expression), reflecting the role of NF1 as an inhibitor of RAS signaling. The first two PCA components together accounted for the majority of variation in protein levels (Figure S2). A cloud plot showing the first two principal components is shown in Figure 1A and revealed three patterns of total and activated protein levels. One component (PC2) distinguished proteins which were correlated with EGFR total protein versus those correlated with PDGFB levels. The other component (PC1) distinguished a third group of proteins anti-correlated with EGFR and PDGFB. Elevated levels of these proteins were associated with low levels of Neurofibromin 1 protein (“NF1 loss” in Figure 1A).

In order to evaluate the significance of this three-way classification, we performed unsupervised k-means analysis on the same standardized data from the 20 GBM samples and evaluated the fit and stability over a range of cluster sizes. K-means for cluster sizes from 2–8 was run for 10,000 iterations, leaving out 15% of the data with each iteration (i.e. three randomly chosen samples left out). Consensus matrices were evaluated for cluster assignment stability both visually and by cophenetic correlation (see Figure S3A). As predicted from PCA, the correlation of protein levels was well-described by 3-way clustering (Figure 1B). Considering only those proteins which co-cluster in >95% of k-means iterations, three “core” protein groups were identified. These protein groups were named according to the strong separation by PCA of EGFR, PDGF and NF1 levels, with consideration that alterations in these three genes are common in glioblastoma and each is pathogenic in genetically-engineered mouse glioma models:

#### EGFR core

This cluster of 15 proteins features high levels of total and phospho-EGFR. Prominent Notch activity is represented in this group by high levels of the active cleaved form of Notch 1 (Notch 1 ICD) as well as of ligands Jagged (JAG1) and Delta-like 1 (DLL1) and of the downstream Notch transcriptional target HES1. Wnt signaling is suggested by increased (stabilized) B-catenin levels. Other proteins relatively increased in this core group are EIF4EBP1, EIF4E, RHEB, phospho-BAD, INI1, La/SSB, FGF2, and phospho-Rb1. There was a trend for higher phospho-Akt levels, though this did not reach significance (p = 0.08).

#### PDGF core

This cluster is comprised of 17 proteins including PDGFB, phospho-PDGFRβ and phospho-NFKB1. PTEN levels are higher in this group and relatively increased Ras activity is evidenced by elevated total and phosphorylated MEK and ERK. Levels of MTOR and downstream targets S6K and p-S6K were better-correlated with the PDGF core than the EGFR core, though levels were high in tumors of both classes. Other PDGF-associated proteins were SHH, HEB 1/2 (TCF12, associated with oligodendrocyte development), BRAF, p-FOXO1 and TSC1.

#### NF1 Core

This group is defined by higher levels of 5 proteins: IRS1, IGFBP5, YKL40 and VEGF. As suggested by PCA analysis, the group is also strongly associated with low levels of NF1 (plotted as the inverse protein level, “NF1 loss”). Sporadic elevation of MYC, NMYC, and KRAS-GTP was also seen in this group. Compared to the other classes, GBM samples showing NF1-core expression pattern showed relatively suppressed levels of total- and phospho-proteins in the PI3K and MAPK pathways. These differences were relative, however, and evaluation of the Western bands shows that high levels of phosphorylation were common in nearly all tumors (see Figure S1).

### Identification of proteomic tumor classes by signaling pattern

**Figure 2:**
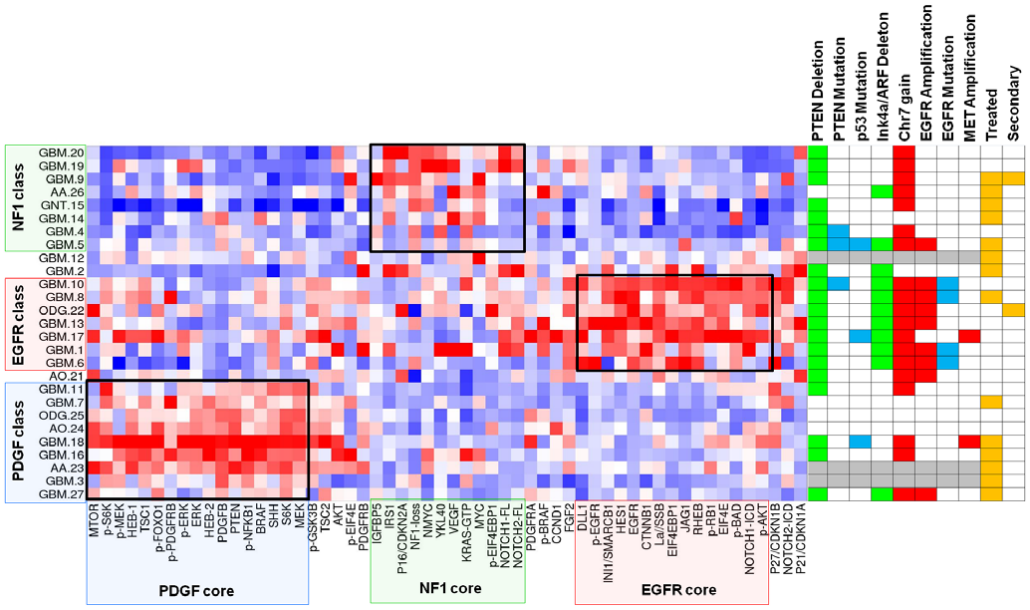
K-means clustering of gliomas by signature-defining proteins. Unsupervised k-means clustering of 27 gliomas by 44 core proteins derived from Figure 1B. 3-way clustering was determined to be the best fit by consensus matrix stability and cophenetic correlation (3). Right: summary of array-CGH, sequencing and clinical information is given for each tumor. Red denotes copy number gain or focal amplification as specified; green marks deletion of at least one copy. Blue denotes mutations (see text). Gray marks samples for which DNA was unavailable. Detailed aCGH profiles shown in Figure S4.

The 44 core proteins identified in the previous analysis were then used to cluster the larger set of 27 glioma samples. K-means clustering of samples was performed as before, leaving out 15% of proteins (7 out of 44) with each of 10,000 iterations. As expected in this supervised approach, three-way clustering was the best fit and all but three glioma samples showed stable clustering into signaling classes associated with EGFR, PDGF and NF1-loss (Figure S3B). Figure 2 summarizes how tumors are clustered by core signature proteins, and how these groups relate to tumor histopathology and genotype derived from aCGH profiling and resequencing of EGFR.

Tumors with GBM histopathology were evenly distributed among the three core groups, six in each group. The only known secondary GBM clustered with the NF1-loss group. Two anaplastic astrocytomas (AA23 and AA.26) were found to cluster disparately with the PDGF and NF1 groups, respectively. Two of the four oligodendrogliomas clustered with the PDGF group: one low-grade (ODG.25) and one anaplastic (AO.24). Interestingly, one of the two low-grade oligodendrogliomas clustered with the EGFR group (ODG.22) and did in fact harbor high-level EGFR amplification. One anaplastic oligodendroglioma (AO.21) showed no distinct proteomic pattern and remained unclassified. There was a trend for untreated tumors in the EGFR tumor group (6 untreated vs. 1 treated) and for treated tumors in the PDGF group (3 untreated, 6 treated) but this was neither significant for GBM (p = 0.11) nor for all tumors (p = 0.09). Testing protein levels individually between treated and untreated GBM, PDGFB was the most significantly different and was higher in treated GBM (p = 0.009) and in treated tumors overall (p = 0.028) although neither was significant after correction for multiple testing.

24 of the 27 tumors for which sufficient tissue was available were analyzed by array-CGH (Agilent Whole Genome 244K) and resequencing of EGFR, PTEN and TP53. Array-CGH profiles were analyzed for copy number aberrations commonly described in GBM: focal amplification of *EGFR*, *MET* and *PDGFRA*; gain of chr7 without focal amplification; loss of chr10 or 10q23 region spanning *PTEN*; loss and/or homozygous deletion of 9p21 including Ink4a/ARF.[1], [23], [24] Figure 2 summarizes the finding of focal amplification of *MET* and *EGFR*, gain of chromosome 7, homozygous deletion spanning the *Ink4a/ARF* locus, and loss at PTEN locus. Complete ACGH profiles are depicted in Figure S4.

#### EGFR proteomic tumor class

Six of the seven tumors in this class showed *EGFR*-region amplification and four showed point mutations in the extracellular domain (ECD) previously described in GBM as activating mutations: A289V (GBM.10 and GBM.6), T263P (GBM.8), G598V (GBM.1) and R108K (GBM.6).[25] The only tumor in the EGFR signaling cluster that did not show focal *EGFR* amplification was found to harbor amplification of a narrow region including *MET*. Neither overexpression nor significant phosphorylation of EGFR protein was seen in this case. All tumors in the EGFR proteomic tumor class had deletion of the *Ink4a/ARF* locus compared with only 3/17 (18%) in the other groups. All had loss of ch10 and mutation of PTEN in the remaining allele was observed in one case.

#### PDGF proteomic tumor class

Although this group of tumors is defined by evidence of PDGF signaling at the protein level, none of the 9 tumors in this class showed gene amplification of either PDGF receptors or ligands. One tumor had both *Ink4a/ARF* region deletion and *EGFR* amplification, however total and phosphorylated EGFR levels in this tumor were low and PDGFB was present. Of note, a second tumor with focal *MET* amplification, GBM.18, was classified here and showed extremely high levels of PDGFB and associated signaling.

#### NF1 proteomic tumor class

Tumors in this class included six GBM, one of which was a secondary GBM, one anaplastic astrocytoma, and one tumor with histopathologic features of GBM and synaptophysin positivity (“glioneuronal tumor”, GNT.15). The NF1-associated class is distinguished by chr7 gain without focal amplification of either *EGFR* or the *MET* receptor which is significantly more frequent in this class (5/7, 71%) compared to the others (2/14, 14%, p = 0.017, Fisher's Exact test).

### Integrated analysis of molecularly defined subclasses of GBM from The Cancer Genome Atlas identifies distinct tumor subclasses enriched for mutations in *EGFR*, *PDGFRA*, and *NF1*

**Figure 3:**
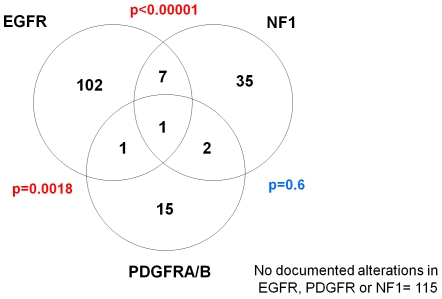
Integration of mutation and chromosomal copy number data from TCGA reveals aberrations of EGFR to be mutually exclusive of aberrations in PDGFRA and NF1. Summary of copy number aberrations (CNA) and mutations of EGFR, PDGFR and NF1 genes in 278 glioblastoma samples from The Cancer Genome Atlas. 163 samples showed mutation/aberration of at least one of the genes. For this summary, only validated, non-synonymous somatic mutations were considered (164/278 samples had sequencing information available). CNA was defined as focal high-amplitude amplification (>4 copies) of EGFR or PDGFR, and by at least single-copy loss of NF1.

Glioblastoma datasets from The Cancer Genome Atlas were analyzed for alterations in *EGFR*, *PDGFRA* and *NF1* and the possible association of mutations in these genes with transcriptomally-defined subclasses. Of 278 tumors for which chromosomal copy number and/or mutation data were available, amplification and/or somatic non-synonymous mutation of *EGFR* was found in 40% (n = 111) and of *PDGFRA* in 7% (n = 19), while chromosomal loss and/or mutation of *NF1* was found in 16% (n = 45). As shown in Figure 3, genomic alterations in these three genes are largely mutually exclusive, suggesting distinct tumor subclasses among this portion of GBM samples for which clear genomic alterations could be detected.

**Figure 4:**
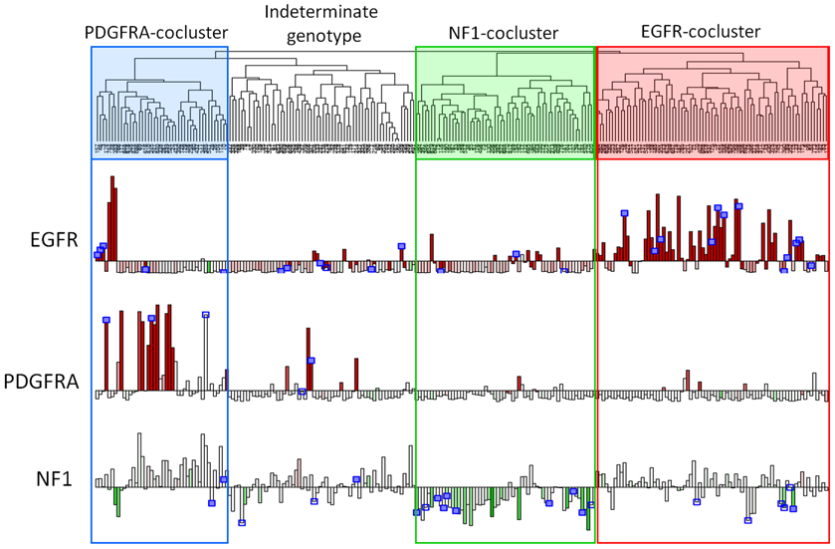
Unsupervised transcriptomal clustering of GBM from the TCGA dataset. Unsupervised hierarchical clustering of gene expression from 243 GBM samples in The Cancer Genome Atlas reveals four transcriptomal clusters, three of which are enriched for alterations of PDGFRA, NF1, and EGFR respectively. Expression data is from Affymetrix U133A and copy number is taken from Agilent 244K platform (TCGA Level 3 public data, see Methods). Gene expression for EGFR, PDGFRA, and NF1 is shown in the bar plots, colored according to gene copy number: amplification (red) or loss (green). Blue boxes denote samples with non-synonymous somatic mutations which have been validated (solid) or are pending validation (open). Three clusters are highlighted which show specific enrichment for lesions in genes encoding key signal transduction pathway members EGFR, PDGFRA and NF1. A fourth cluster lacks clear enrichment for any specific mutation or CNA.

We next sought to determine if these subclasses might be associated with distinct transcriptomal signatures. Using normalized gene expression profiles on 243 tumors downloaded from The Cancer Genome Atlas public portal, we applied unsupervised hierarchical clustering which identified 4 main cluster branches (Figure 4). This grouping was concordant with comparable 4-way cluster structure recently reported by the TCGA.[26] Integration of copy number aberration, mutation and expression of *EGFR*, *PDGFRA* and *NF1* revealed that three of the transcriptomal classes were enriched for alterations in each gene, respectively, although the segregation was imperfect. We named these three subclasses “EGFR cocluster”, “PDGFRA cocluster” and “NF1 cocluster” based on the predominant signal transduction pathway member enriched for mutation in each group. A fourth transcriptomal class had neither significant enrichment for mutations in either of *EGFR*, *PDGFRA* or *NF1*, nor enrichment for mutations in any other sequenced gene in the TCGA dataset. As shown in Figure 4 as described below, *PDGFRA* amplification was only present in the minority of PDGFRA-cocluster tumors and this transcriptomal class included a subset of tumors with EGFR or Met amplifications. The PDGFRA-cocluster showed high expression of *GRIA2*, *OLIG2*, and *NCAM1/2* as well as other genes which are signatures of the “Proneural” transcriptomal class of GBM previously described.[19] Conversely, signature genes of the “Mesenchymal” GBM class such as *YKL40/CHI3L1*, *IGFBP2* and *VEGFA* were most highly expressed in the NF1-cocluster transcriptomal group (Figure S5). The EGFR-cocluster class showed intermediate expression levels of Proneural and Mesenchymal signature genes.

Unsupervised clustering reflects the combined effect of multiple confounding influences on the transcriptome, including presence of necrosis, inflammatory cells, inclusion of brain parenchyma, etc. In order to clarify the specific relationship of *EGFR*, *PDGFRA* and *NF1* mutations to the transcriptome in GBM, we derived distinct expression signatures associated with mutation/aberration of these genes among the set of 147 tumors for which mutation/aberration was found in one and only one of the three. Together, 1,943 genes were identified which distinguished the three mutation classes (see Methods). Hierarchical clustering performed with this gene set on the full set of 243 GBM profiles resulted in clear division of GBM into just 3 subclasses, each enriched for the associated alteration of *EGFR*, *PDGFRA* and *NF1* (Figure S6).

### Comparison of proteomic and transcriptomal subclasses

We investigated possible correspondence between the proteomic EGFR, PDGF and NF1 classes and the transcriptomal EGFR-, PDGFRA-, and NF1-coclusters derived from TCGA data by comparing signature genomic aberrations. As shown in Figure 4, integration of mutation and copy number data confirmed the strong association of *EGFR* amplification and mutation in tumors from the EGFR-cocluster (59/79, 75%) significantly more than in the other two classes (21/99, 21.2%, p = 7.8e−13). This subset of samples also harbored frequent homozygous deletion of 9p21 spanning the *Ink4a/ARF* locus (75% vs 35%, p = 2.0e−7). These findings were concordant with genomic profiling of tumors the EGFR signaling class. Conversely, chr7 gain without focal amplification of either *EGFR* or *MET* was most common in the NF1 transcriptomal cocluster (60.7% vs 23%, p = 1.6e−6), and this was concordant with aCGH profiling of tumors in the NF1 signaling class.

Among TCGA samples, amplifications of *PDGFRA* were more commonly seen in the PDGFRA transcriptomal cocluster (25.6% vs 1.5% in other classes; p = 2.9e−6) as were *PDGFRA* mutations (3/29 = 10.3% vs 0/79, p = 0.049). However 4 out of 5 *MET*-amplified tumors were in this class and 7 tumors showed focal amplification of *EGFR*. Therefore there was no single copy number aberration distinguishing this class. There were no cases of *PDGFRA* amplification among the 24 tumors in our study for which aCGH was performed. Among the 9 tumors in our PDGF signaling class, one had *MET* amplification and one had *EGFR* amplification. Despite the absence of *PDGFRA*-amplified cases in our sample set, the overall distribution of *EGFR*, *PDGFRA* and *MET* amplifications was found to be within the normal sampling error if one derives expectation frequencies from the TCGA data.

Comparison of genomic aberrations suggested a correspondence between proteomic classes of EGFR, NF1 and PDGF and the corresponding TCGA transcriptomal classes, therefore expression levels for genes encoding the core proteins (total forms) were next assessed in each of the four transcriptomal clusters. Among genes encoding EGFR-core proteins, the corresponding EGFR transcriptomal cocluster subclass showed significant overexpression of *EGFR*, *JAG1*, and *HES1* compared to the other subclasses (p<1e−5 for each gene). However other EGFR core proteins and Notch pathway members showed no elevation of mRNA expression in the corresponding transcriptomal class and therefore direct correlation could not be established.

Within the transcriptomal NF1-cocluster, the only overexpressed genes encoding NF1 core proteins were *IRS1* and *YKL40/CHI3L1*. As seen in Figure 4, NF1 mRNA was strongly underexpressed in the NF1-cocluster class compared to other classes (p = 4.4e−15), making *NF1* mRNA underexpression among the strongest signatures of the transcriptomal class.

Within the PDGFRA transcriptomal cocluster, *TSC2* and *HEB/TCF12* were the only genes among the PDGF core protein group significantly overexpressed (p<1e−4). The *PDGFRB* gene was significantly *underexpressed* in this group, concordant with our observation that total protein levels were higher in the EGFR class even while the phosphorylated receptor was correlated with the PDGF class. Overexpression of *PDGFRA* was a prominent feature of the PDGFRA-cocluster tumors (p<1e−12), nearly always associated with gene amplification. Messenger RNA levels of genes encoding PDGF ligands were not elevated in the PDGFRA cocluster even among the subset of tumors showing *PDGFRA* amplification. We further investigated the relationship of PDGFB mRNA and protein levels in a validation set of 40 gliomas and found no correlation between mRNA expression and levels of protein even though the latter were highly variable (Figure S7). This reflects the strong regulation of PDGF at the level of translation rather than transcription.[27], [28] It is likely that some signaling proteins in our study are closely coupled to mRNA levels while many others are regulated independently (or in negative feedback) with corresponding mRNA.

## Discussion

Given the importance of signaling in the biology of gliomas, dividing these tumors into subsets by the pattern of coordinate signaling pathway activation may have practical implications for choice of therapies and for interpretation of patient responses in existing clinical trials. In order to clarify the net activation of signaling pathways we used a targeted proteomic analysis to determine not only the levels but the posttranslational modifications associated with signaling activity. The intrinsic cellular heterogeneity of gliomas is masked by the methods used in this study since both the proteomic and TCGA genomic analyses are performed on homogenized tissue, blending the characteristics of the cells together. Additionally, the small sample size and selection of proteins in this study limits the statistical power to define protein correlations and sample assignments. Nonetheless, we find common features defining three basic groups by both genomic and protein analysis, illustrating the high complementarity between protein signaling activity, transcriptomal signature and genomic alteration in GBM. The observed enrichment of *EGFR*, *PDGFRA* and *NF* genomic alterations with transcriptome pattern could mean that signaling activity directly influences the transcriptome and/or that both signaling and the transcriptome patterns are part of a common underlying phenotype. Comparing unsupervised and supervised clustering results, it is likely that only a portion of the transcriptomal features distinguishing unsupervised clusters are associated with signaling, either directly or indirectly. In fact, unsupervised clustering identifies four clusters and broader phenotypic and genetic differences distinguishing these four transcriptomal groups have been reported.[26]

Analysis of the downstream signaling components of the PDGF proteomic group revealed generally lower PI3K/Akt activity than in the EGFR glioma group although S6 phosphorylation was paradoxically high. Histologic analysis of the tumors provided an explanation by demonstrating that strong pS6 immunopositivity was localized in reactive astrocytes rather than tumor cells per se (data not shown), concordant with recent observations of mTOR activation in reactive astrocytes under experimental conditions of injury.[29] These cells were more common in PDGF-class tumors. We found a trend for treated tumors to be in the PDGF proteomic class and PDGFB levels were significantly higher in treated compared to untreated tumors. It is possible that some of the features of the PDGF signaling pattern are influenced by prior treatment though it is unlikely that this accounts for the genotypic differences in this proteomic tumor class, such as the paucity of EGFR amplification, chr7 gain and Ink4a/ARF locus deletion. Comparison with treated samples in TCGA is complicated by the fact that the current dataset contains few treated cases and of these, many are secondary GBM which would arguably be assigned to the PDGFRA co-cluster by their common Proneural signature.

The histology of the TCGA samples was uniformly GBM, but this is a histologically heterogeneous tumor type. A priori, it is possible that the transcriptomal classes identified in this analysis could be related to tumor sampling or microenvironment. However, this does not appear to be the case since well-defined genetic lesions are enriched in specific tumor classes and there is no evidence for regional localization of mutations in glioma as a general phenomenon. Although clinical and pathologic data are limited and a more detailed review of this information is underway, there appeared little clinical or histological differences between the three groups identified in this analysis. PDGFRA-cocluster tumors in TCGA occurred in younger patients, and there were small but significant differences in the amount of associated necrosis and inflammatory cells (data not shown).

It remains to be established whether ligand-driven PDGF signaling is common among tumors in the transcriptomal PDGFRA-cocluster and whether this is functionally important. We have shown that PDGFB ligand levels are highly variable in GBM, are associated with receptor activation, and are not correlated with mRNA expression. The PDGFRA-cocluster transcriptomal class shares features with the Proneural group of gliomas identified by Phillips et al using transcription analysis, and is characterized by genes expressed during normal cortical oligodendrocyte development such as olig2, Sox2 and doublecortin and signaling pathways involved in that process as well, such as PDGF and SHH. While the PDGFRA-cocluster group is enriched for Proneural signature genes, it is important to note that the original signature was derived from a dataset of mixed histologies and the analysis designed specifically to resolve a prognostic signature. Therefore the exact relationship between our PDGF proteomic class, the PDGFRA co-cluster, and gliomas harboring Proneural signature is unclear and will need to be further investigated.

Although *PDGFRA* amplification predominated in the PDGFRA-cocluster transcriptomal group, a full 15% showed amplified *EGFR* and another 15% showed amplified *MET*. From the prevalence of PDGF signaling we found at the protein level one might hypothesize the existence of concurrent PDGF signaling in *EGFR*- and *MET*- amplified tumors in this class. In fact, we found two such tumors in our proteomic analysis: one *EGFR*- and one *MET*-amplified, both with high levels of PDGFB, phosphorylation of PDGFRβ and an overall signaling pattern matching the PDGF proteomic class. It is unclear in these cases whether the level of PDGF pathway activation is functionally important, perhaps in a subpopulation of cells. It is notable that 6 tumors in TCGA show focal amplification of both *PDGFRA* and another RTK: four cases sharing focal co-amplification of *EGFR*, and two cases sharing focal co-amplifications of *PDGFRA* and *MET*.

In conclusion, our findings support a division of GBMs into three classes according to patterns of signal transduction pathway activation. These patterns reflect, in part, mutually exclusive signaling involving EGFR, PDGF RTK activation or NF1 silencing. Both the transcriptomal and proteomic classes were imperfectly related to genotype, suggesting that molecular assays used in patient stratification and clinical trial analysis should include measures of PDGF ligand and receptor phosphorylation as well as NF1 expression. Notch signaling was prominently associated with the EGFR class at the protein level, an observation which was not predicted by mRNA expression levels of Notch pathway members in EGFR-altered tumors from TCGA. Whether one or more non-EGF/PDGF RTKs are contributing to NF1 tumors is uncertain, but the finding that NF1-silenced tumors show elevated MET, HGF and IRS1 at the transcriptomal level and validation of IRS1 at the protein level suggest IGF and or MET signaling may be contributory. Further refinement of GBM subclasses will likely come from direct investigations of these and other signaling proteins, as well as investigation of newly described recurrent mutations in GBM such as ERBB2 and IDH1. The current study provides an initial architecture for such subclasses and suggests the potential for class-directed therapies.

## Methods

### Ethics statement

The collection and use of the human tissues in this study were performed after obtaining written consent from all participants, in accordance with a study protocol approved by the Institutional Review Board of Memorial Sloan-Kettering Cancer Center. Data from The Cancer Genome Atlas public portal were obtained under an approved Data Access Request.

### Surgical glioma sample analysis

#### Tumor samples

Tumors were snap-frozen in the operating room, and stored at −80°C. Samples in liquid nitrogen were ground to powder and protein was extracted through lysis with T-per tissue extract solution (Pierce) supplemented with 30 mM sodium fluoride, 1 mM sodium vanadate, and protease inhibitor cocktail tablets (Roche). Protein concentrations were determined by bicinchoninic acid assay (BCA) method (Bio-Rad).

#### Western blot analysis

Samples (100 µg) were separated by 6, 8, 10, or 12% SDS-PAGE gel, and transferred onto polyvinylidene difluoride membrane (Millipore). For qualitative comparison, analysis included normal brain cortex lysate as previously described (Analytical Biological Services Inc).[30] Membranes were blocked with 5% nonfat milk in PBS-0.1% Tween 20. Primary and secondary antibodies were diluted in the blocking solution. Signal was visualized using enhanced chemiluminescence (Amersham Biosciences). Primary antibodies used in this study are listed in Table S1. Secondary peroxidase-conjugated anti-rabbit antibody (Amersham Biosciences), anti-mouse and anti-goat antibodies (Roche) were used at 1∶1,000 dilution. For NF1, 50 ug of lysate was run on 6% gel and secondary antibody was 1∶10,000 dilution. PDGFRα total protein was assayed at a later date by western blot of frozen banked lysate aliquots (Santa Cruz, #sc-338, 1∶500, secondary 1∶10,000). Because of low band intensity a second PDGFRα antibody was tested as well and gave concordant results (Cell Signaling #3174, 1∶1000, secondary 1∶2,000). The density of each band was read and quantified using Adobe Photoshop and NIH Image 1.63 software and normalized by actin control. PDGFRα western was normalized to tubulin.

#### Activated ras pull-down assay

Activated K-ras (K-ras-GTP) was tested by using 500 µg samples for activated Ras pull-down with 10 µg of glutathione-conjugated Raf-1 GST-RBD beads (Upstate Biotechnology) as previously described.[31]

#### Unsupervised clustering of protein data

Quantified western data were standardized for each protein by mean and standard deviation across the sample cohorts. Standardization was done first on the glioblastoma samples (n = 20) for unsupervised clustering analysis of protein level, then again separately on the whole sample set (n = 27) for cluster analysis of samples. For the purpose of display, NF1 is represented as “NF1-loss”, or zero minus standardized NF1 quantity on western. All statistical analyses and plotting were done in R (www.cran.org). Principal component analysis was performed on standardized data. K-means clustering of quantified protein levels for 20 GBM samples was performed in R, and stability of cluster assignment assessed over 10,000 iterations leaving out 15% of tumors with each iteration (kmeans, stats package, www.cran.org). Consensus matrices were generated for each k-clustering over all iterations and assessed visually as well as by cophenetic correlation. Core correlated protein clusters are defined for 3-way clustering as >95% consensus. Proteins in these core clusters were selected for k-means analysis of 27 gliomas using, as before, an 85% resampling of proteins and consensus matrix analysis over 10,000 iterations.

#### ACGH and resequencing

Genomic DNA was extracted from primary tumors using standard techniques. DNA was then digested and labeled and hybridized to 244K CGH arrays according to manufacturer guidelines (Genomic DNA labeling kit PLUS, Agilent). This array consists of >238,000 coding and non-coding sequences allotted to assembly map positions (NCBI, Build 35). Normal male genomic DNA (Promega, Madison, WI) was used as a reference. After washing, the slides were scanned with an Agilent scanner and images quantified using Feature Extraction 9.5.3.1 (Agilent). Fluorescence ratios of the scanned images were calculated and the raw aCGH profiles were processed to identify statistically significant transitions in copy number using the Circular Binary Segmentation algorithm.[32] Each profile was centered so that log2 ratio of zero is assigned to the predominant copy number, determined by the mode of the distribution of the mean log2 ratio for each segment, weighted by the number of probes per segment . After mode-centering, gains and losses for a subset of analyses were defined as segment mean log2 ratios of >0.2 or <−0.2 and amplification and deletions as >2 or <−1, respectively. Additionally, sample-specific thresholds for alterations were computed for all other analyses. The annotated microarray data for the sample set is available on GEO (www.ncbi.nlm.nih.gov/geo, GSE17381).

### Integrated analysis of genomic data from The Cancer Genome Atlas

#### Dataset compilation and analysis

A description of TCGA data types, platforms and analyses are as previously described.[8] Processed genomic datasets were downloaded from The Cancer Genome Atlas public data portal (http://cancergenome.nih.gov/dataportal/) as available on May, 2009. Specific data sources were as follows: For mRNA expression, “Level 3” normalized gene expression derived from the Cancer Genome Characterization Center (CGCC) at the Broad Institute, MIT (Affymetrix Human Genome HTS U133A 2.0). For chromosomal copy number, “Level 3” normalized and segmented copy number data from the CGCC at Memorial Sloan-Kettering Cancer Center (Agilent 244K CGH Array). From this array-CGH dataset one unique profile was selected for each tumor based on the highest signal-to-noise estimate. For sequencing data, we combined all available sequencing data summaries in “multiple alignment format” (MAF) files as of May 2009: broad.mit.edu GBM.ABI.1, genome.wustl.edu GBM.ABI.53, hgsc.bcm.edu GBM.ABI.1.maf and GBM.ABI.2. Mutations were further filtered by excluding events which were classified as “somatic,” “synonymous” or “silent,” or “unvalidated,”

A list of the sample IDs and the summary of genomic data are given in Table S3. 243 samples had gene expression from Affymetrix U133A platform. Of these, 237 also had aCGH data and 159 had mutation data available. Level 3 array-CGH data was used for local copy number estimation. Amplification was defined by regional log2 ratio >2.0. Gene loss was classified by minimal log2 ratio (log2R) across the gene as follows: “single copy loss” = −1.0<log2R<−0.2; “homozygous deletion” = log2R<−1.0.

#### Clustering of TCGA expression dataset

Unsupervised clustering of TCGA Level 3 expression data was performed on 1,807 genes representing the top 15%ile of variance (hierarchical clustering, correlation metric, complete linkage; hclust, R package:stats,). Supervised cluster analysis was performed as follows: 147 samples were identified harboring only one of either EGFR mutation/amplification (n = 103), PDGFRA mutation/amplification (n = 14) or NF1 mutation/loss (n = 30). Kruskal-Wallis (kruskal.test, R package:stats) test was used to identify genes which discriminate between EGFR-, PDGFRA- and NF1-altered samples. Because only 14 samples in TCGA set harbor solitary PDGFRA alteration, the numbers of profiles in each class are balanced by sampling 14 of the 103 EGFR-altered and 14 of the 30 NF1-altered samples and the KW test is run iteratively 1000 times with resampling. 1,953 genes were found to show KW p-values <0.05 in >95% of iterations and these were used to perform supervised hierarchical clustering. Calculation of significance for differences between cluster was by Fisher's Exact Test (R package:stats) using cluster assignments for PDGFRA, NF1 and EGFR-coclusters derived from unsupervised clustering, and excluding samples belonging to the “Indeterminate Genotype” cluster.

## Supporting Information

Figure S1 Selected bands from western blots which were quantified in this study. Signaling class assignments are shown for those samples with stable clustering: “P” =  PDGF class, “N” = NF1 class, and “E” = EGFR class.
(6.30 MB TIF)

Figure S2 Principal Componenet Analysis of Quantified Protein Levels: Fractional variance for principal components from the analysis of quantified protein levels in 20 GBM samples. The first two components together account for 51% of total variance and are plotted in Figure 1A.
(0.34 MB TIF)

Figure S3 Analysis of stable cluster assignment by k-means for varying cluster count: K-means clustering of quantified and standardized protein levels in GBM; classification of gliomas by core protein expression patterns. For each analysis, K-means was run for 10,000 iterations leaving out 15% of data with each iteration. Shown are consensus matrices for division into 2–4 clusters, and cophenetic correlations for division into 2–8 clusters. (A) Clustering of 55 proteins in 20 GBM samples shows stable two-way and three-way clustering with peak cophenetic correlations ∼0.98. Details for 3-way clustering are shown in Figure 1B in the main text. Three sets of “core” proteins (n = 46 total) are defined by their stable cluster membership in >95% of iterations. (B) 27 glioma samples clustered by 44 core proteins identified in the preceding analysis and highlighted in Figure 1B. Gliomas are classified into 3 types based on the levels of 44 total and activated protein forms.
(1.04 MB TIF)

Figure S4 Genomic profiling of gliomas clustered by signaling class: Array-CGH shows high concordance between EGFR signaling class and amplification of EGFR locus and deletion of the Ink4a/ARF locus. Tumors in the NF1 class show frequent gain of chr7 without focal amplification of either EGFR or MET. Of the two tumors which do have focal MET amplification, one clusters with EGFR-class and the other with PDGF-class. No amplification of PDGFRA was found in any of the samples.
(0.96 MB TIF)

Figure S5 Expression of Proneural and Mesenchymal signature genes in transcriptomal subclasses: Expression analysis of Proneural and Mesenchymal signature genes across transcriptomal subclasses derived from The Cancer Genome Atlas. Unsupervised clustering of TCGA samples and subclass assignments are as shown in Figure 4. Tumor profiles in each subclass are assessed for enrichment of signature genes defining the Proneural and Mesenchymal transcriptomal classes of GBM previously described [19]. Box plots show the distribution of mean percentile rank for expression of Proneural and Mesenchymal signature gene sets courtesy of Kenneth Aldape, MD: Proneural =  BMP2, GRIA2, OMG, NCAM1&2, OLIG2, BCAN, RTN1, SNAP91, GABBR1&2, and KCNB1; Mesenchymal =  YKL40/CHI3L1, IGF2BP3, VEGFA, COL1A1, COL5A2, COL3A1.
(0.35 MB TIF)

Figure S6 Supervised transcriptomal clustering of GBM tumors in The Cancer Genome Atlas: Clustering of 243 GBM samples form TCGA using ∼1,900 genes selected for their ability to discriminate three genotypes: EGFR mutation/amplification, PDGFRA mutation/amplification or NF1 mutation/deletion (see Methods). Sample set and figure legend are as shown in Figure 4 and clustering methods differ only in the subset of genes used. Samples are clustered into three divisions each enriched for one of the three genotypes.
(0.91 MB TIF)

Figure S7 PDGFB protein levels are not correlated with mRNA expression: PDGFB protein levels were assessed in a validation panel of 40 gliomas by western blot and compared with mRNA expression levels. Although the ligand is expressed at highly variable amounts there is no correlation with mRNA, concordant with post-transcriptional regulation of PDGF.
(0.77 MB TIF)

Table S1 Antibodies and conditions used for western blot panel. Antibody sources and conditions as shown. *Hes1 antibody kindly provided by Dr. Tetsuo Sudo (Toray Scientific, Japan).
(0.03 MB XLS)

Table S2 Quantified western results. Western bands quantified by densitometry and normalized to actin (see Methods). Images of selected bands are shown in Figure S1.
(0.05 MB XLS)

Table S3 Summary of integrated analysis of genomic data from 278 samples in The Cancer Genome Atlas. In this table, mutations are denoted in blue and designated “validated” and “unvalidated” according to whether TCGA reports that the mutation was verified by second sequencing method or whether such verification is pending. Focal amplifications in red and single-copy loss or homozygous deletion (light and dark green, respectively) are inferred from array-CGH log2 ratios (see Methods). Cluster assignments are derived from Figure 4.
(0.08 MB XLS)
