# Supplementary figures and images for: Glioblastoma Subclasses Can Be Defined by Activity among Signal Transduction Pathways and Associated Genomic Alterations
Source: PLoS One. 2009 Nov 13;4(11):e7752. doi: 10.1371/journal.pone.0007752 (PMC2771920; doi:10.1371/journal.pone.0007752)

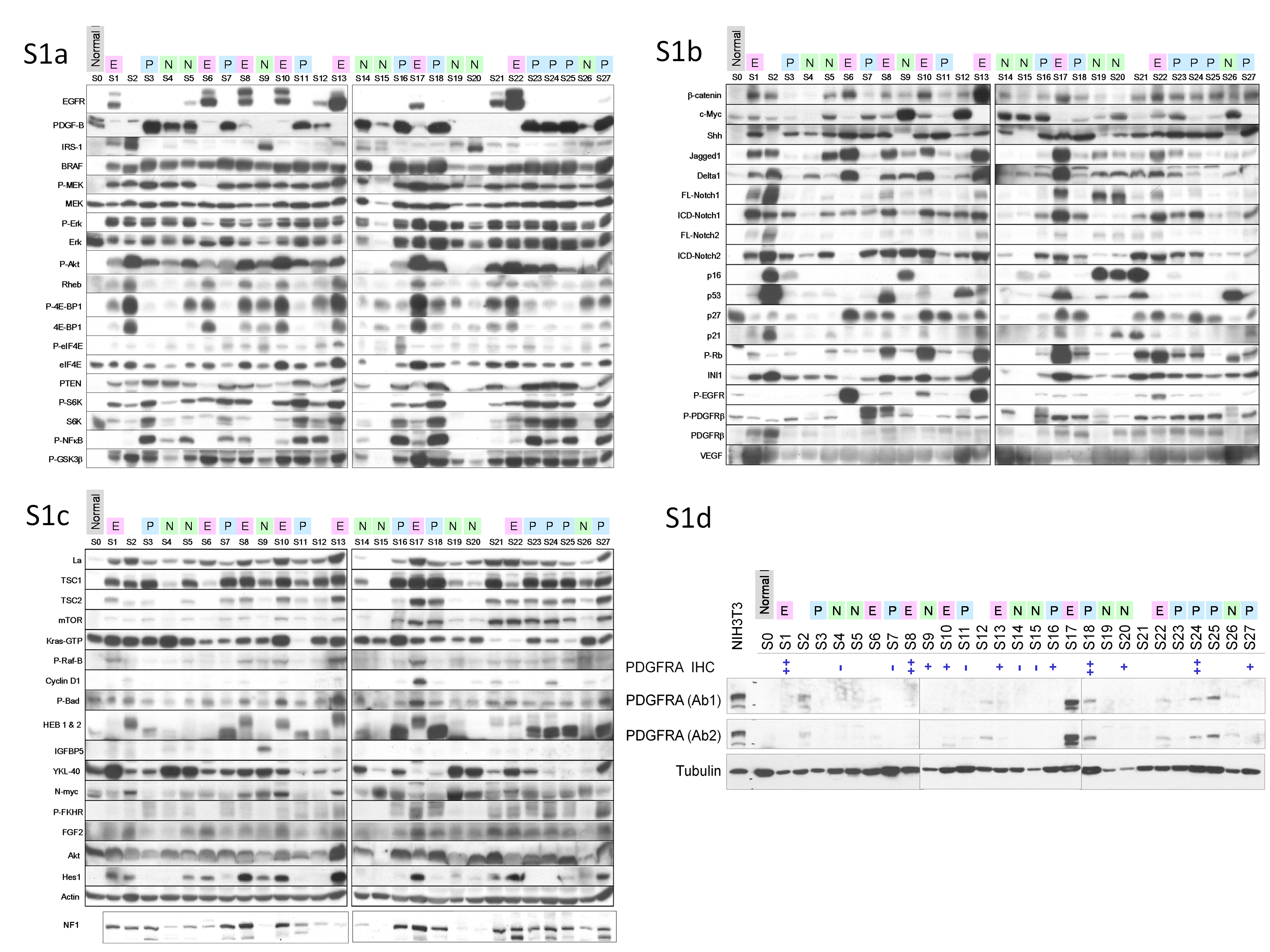

Supplement: Figure S1 — Selected bands from western blots which were quantified in this study. Signaling class assignments are shown for those samples with stable clustering: “P” = PDGF class, “N” = NF1 class, and “E” = EGFR class. (6.30 MB TIF) [file pone.0007752.s001.tif]

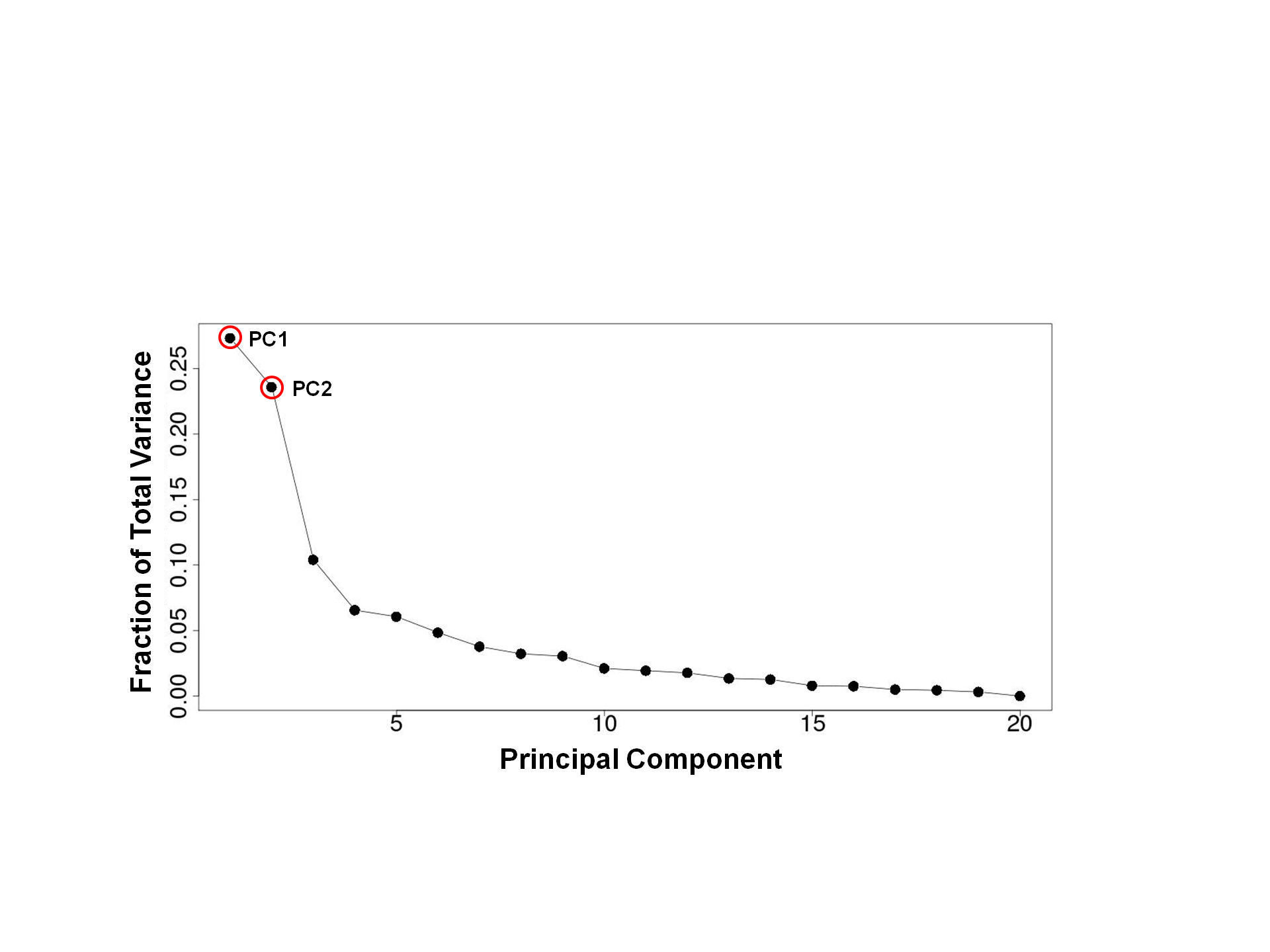

Supplement: Figure S2 — Principal Componenet Analysis of Quantified Protein Levels: Fractional variance for principal components from the analysis of quantified protein levels in 20 GBM samples. The first two components together account for 51% of total variance and are plotted in Figure 1A. (0.34 MB TIF) [file pone.0007752.s002.tif]

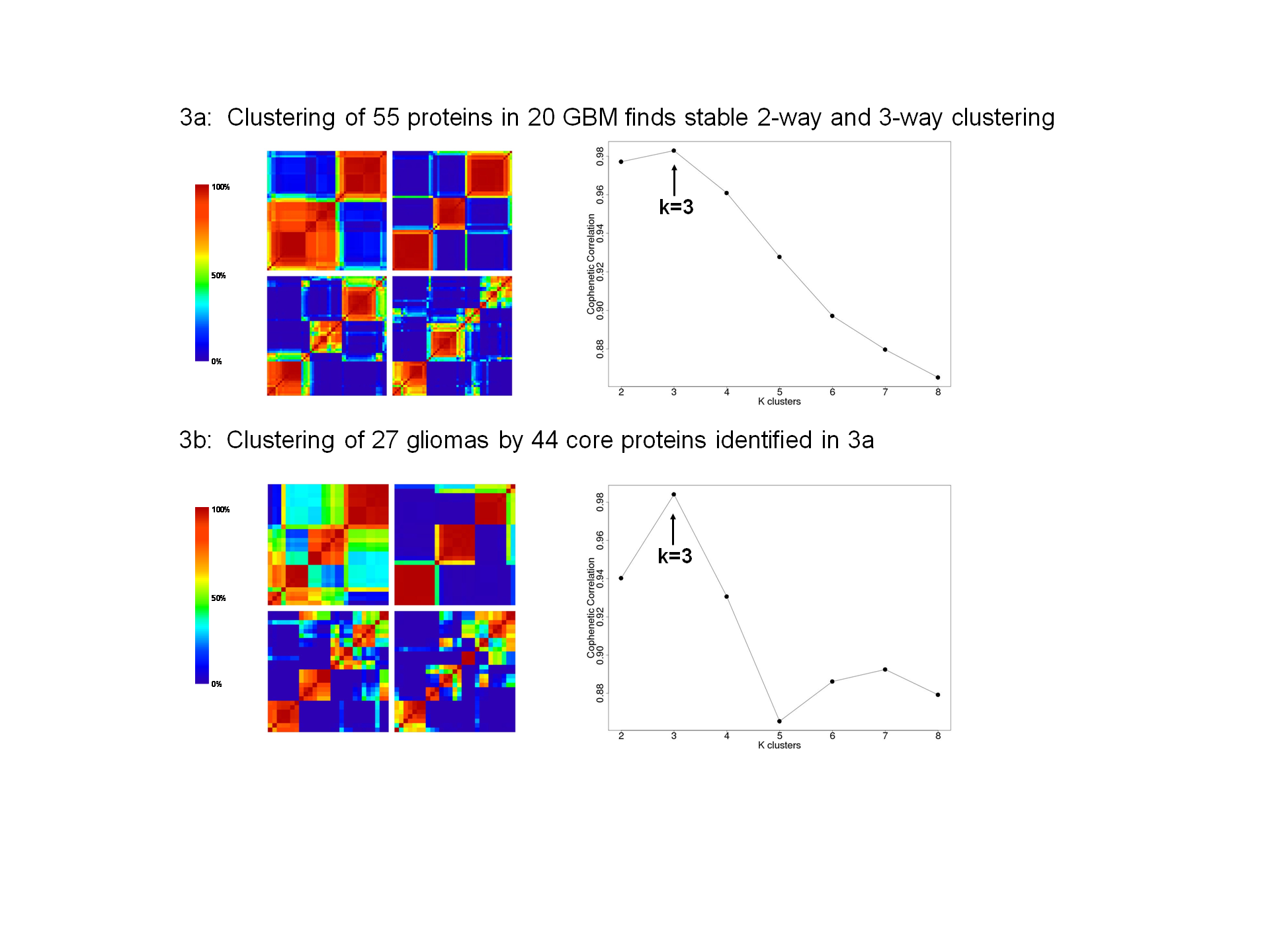

Supplement: Figure S3 — Analysis of stable cluster assignment by k-means for varying cluster count: K-means clustering of quantified and standardized protein levels in GBM; classification of gliomas by core protein expression patterns. For each analysis, K-means was run for 10,000 iterations leaving out 15% of data with each iteration. Shown are consensus matrices for division into 2–4 clusters, and cophenetic correlations for division into 2–8 clusters. (A) Clustering of 55 proteins in 20 GBM samples shows stable two-way and three-way clustering with peak cophenetic correlations ∼0.98. Details for 3-way clustering are shown in Figure 1B in the main text. Three sets of “core” proteins (n = 46 total) are defined by their stable cluster membership in >95% of iterations. (B) 27 glioma samples clustered by 44 core proteins identified in the preceding analysis and highlighted in Figure 1B. Gliomas are classified into 3 types based on the levels of 44 total and activated protein forms. (1.04 MB TIF) [file pone.0007752.s003.tif]

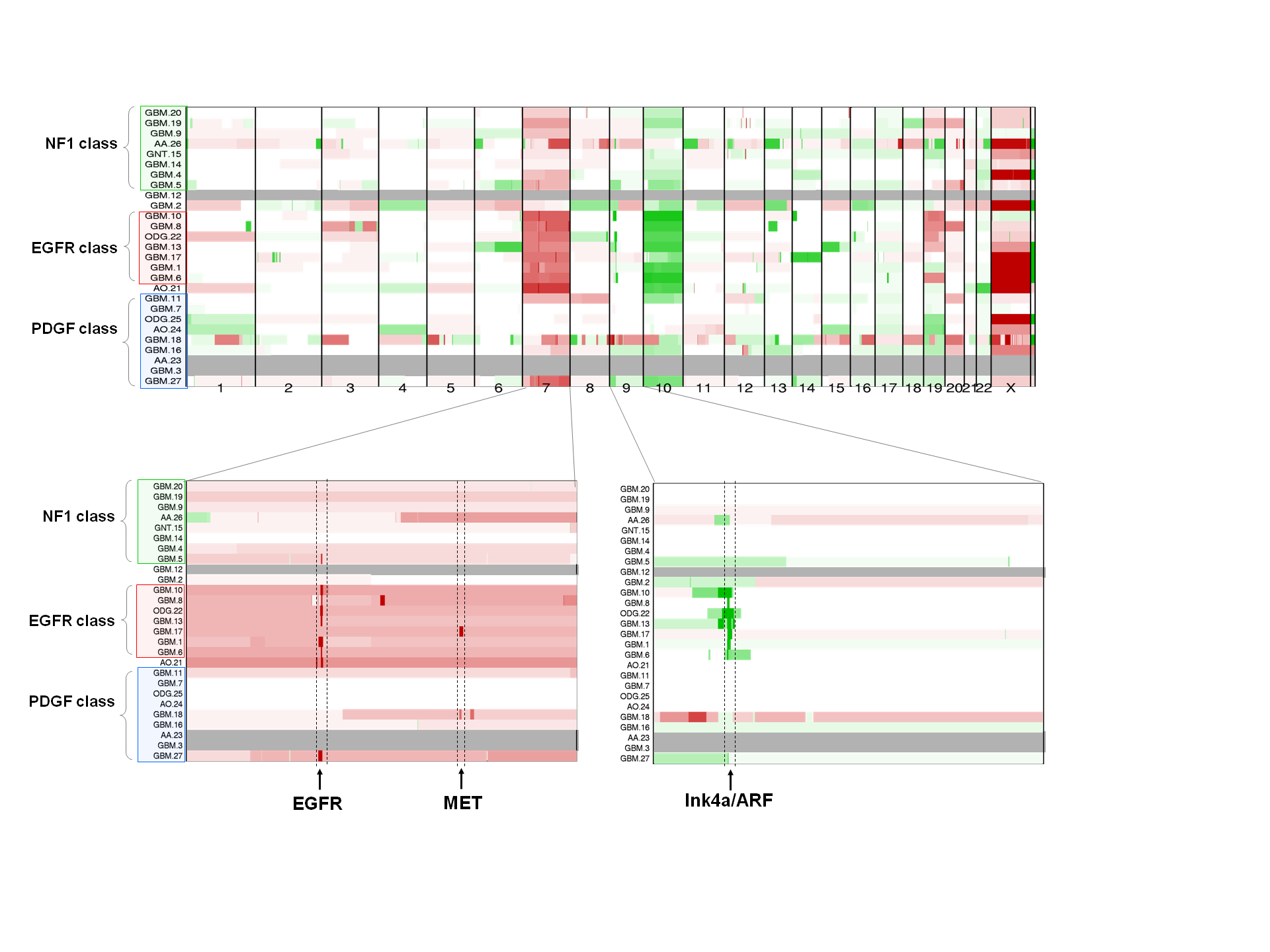

Supplement: Figure S4 — Genomic profiling of gliomas clustered by signaling class: Array-CGH shows high concordance between EGFR signaling class and amplification of EGFR locus and deletion of the Ink4a/ARF locus. Tumors in the NF1 class show frequent gain of chr7 without focal amplification of either EGFR or MET. Of the two tumors which do have focal MET amplification, one clusters with EGFR-class and the other with PDGF-class. No amplification of PDGFRA was found in any of the samples. (0.96 MB TIF) [file pone.0007752.s004.tif]

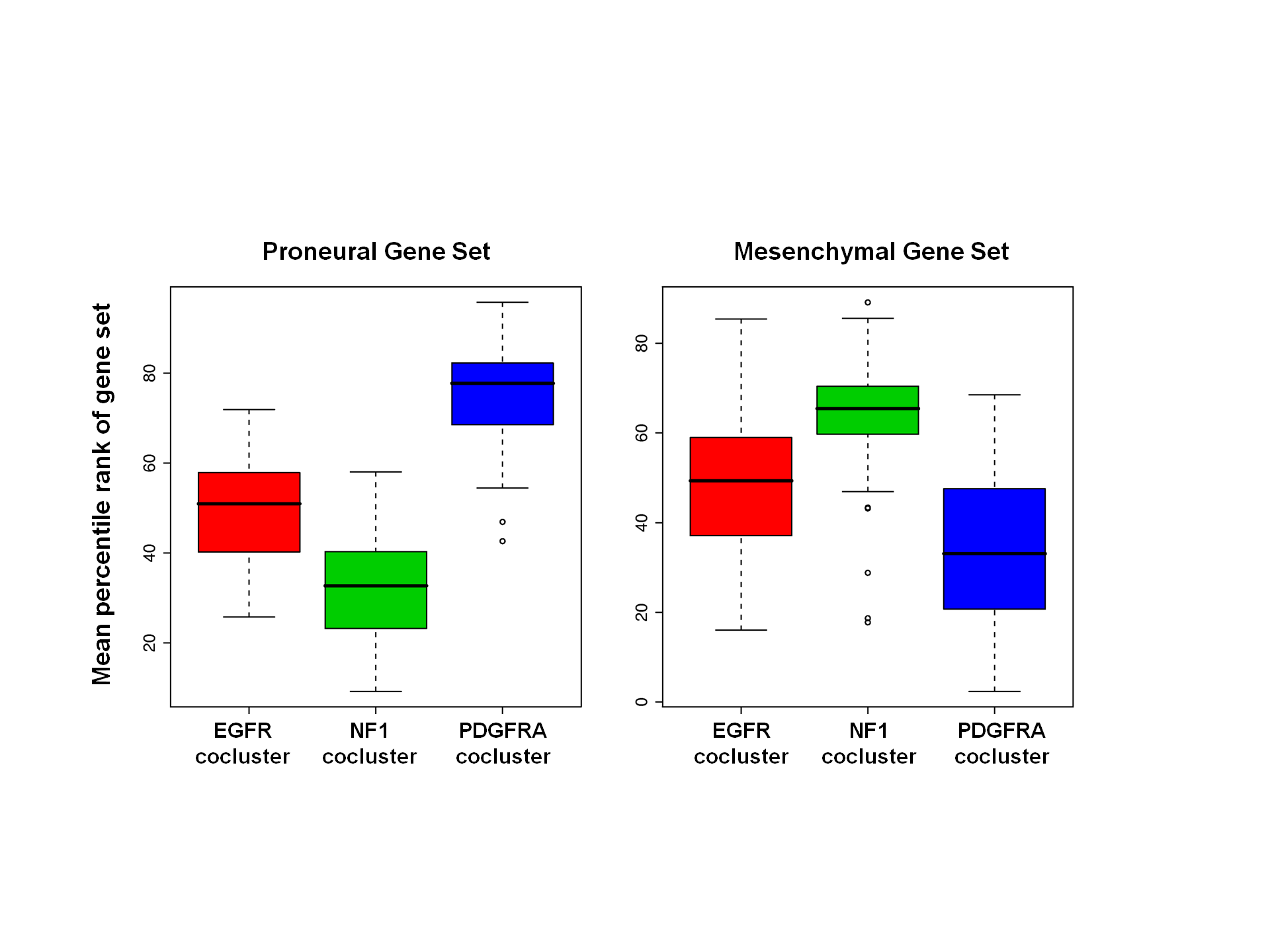

Supplement: Figure S5 — Expression of Proneural and Mesenchymal signature genes in transcriptomal subclasses: Expression analysis of Proneural and Mesenchymal signature genes across transcriptomal subclasses derived from The Cancer Genome Atlas. Unsupervised clustering of TCGA samples and subclass assignments are as shown in Figure 4. Tumor profiles in each subclass are assessed for enrichment of signature genes defining the Proneural and Mesenchymal transcriptomal classes of GBM previously described [19]. Box plots show the distribution of mean percentile rank for expression of Proneural and Mesenchymal signature gene sets courtesy of Kenneth Aldape, MD: Proneural = BMP2, GRIA2, OMG, NCAM1&2, OLIG2, BCAN, RTN1, SNAP91, GABBR1&2, and KCNB1; Mesenchymal = YKL40/CHI3L1, IGF2BP3, VEGFA, COL1A1, COL5A2, COL3A1. (0.35 MB TIF) [file pone.0007752.s005.tif]

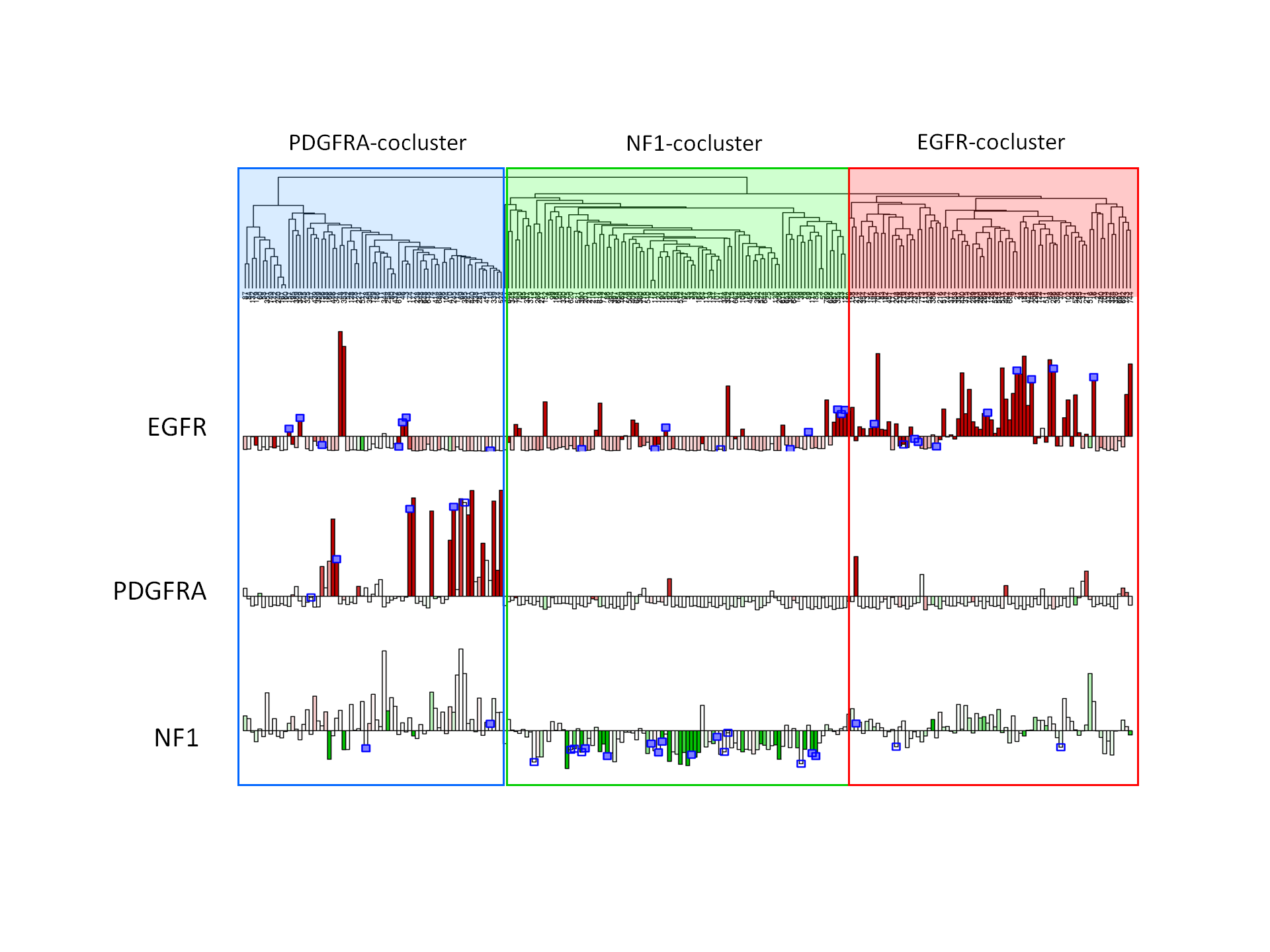

Supplement: Figure S6 — Supervised transcriptomal clustering of GBM tumors in The Cancer Genome Atlas: Clustering of 243 GBM samples form TCGA using ∼1,900 genes selected for their ability to discriminate three genotypes: EGFR mutation/amplification, PDGFRA mutation/amplification or NF1 mutation/deletion (see Methods). Sample set and figure legend are as shown in Figure 4 and clustering methods differ only in the subset of genes used. Samples are clustered into three divisions each enriched for one of the three genotypes. (0.91 MB TIF) [file pone.0007752.s006.tif]

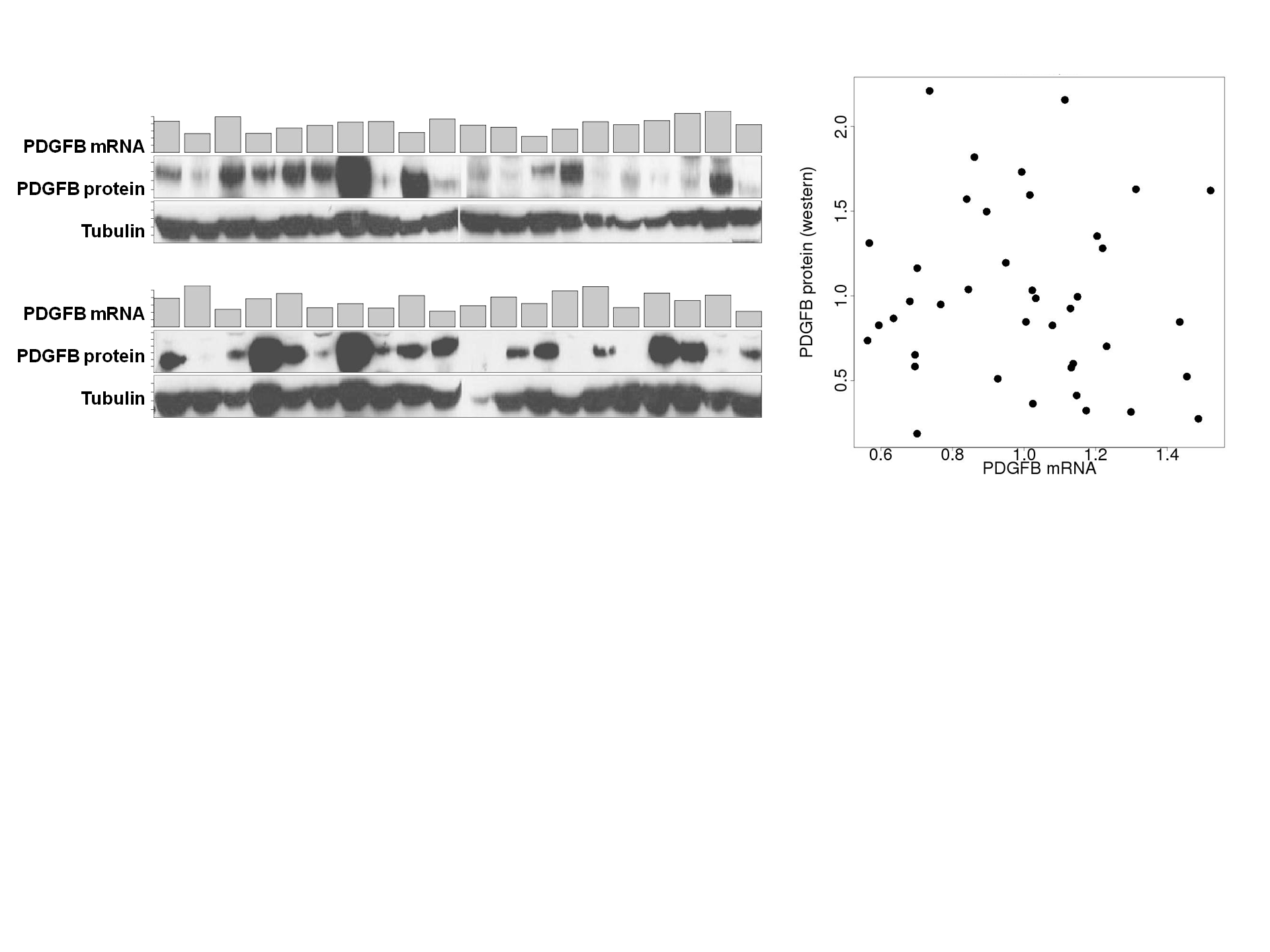

Supplement: Figure S7 — PDGFB protein levels are not correlated with mRNA expression: PDGFB protein levels were assessed in a validation panel of 40 gliomas by western blot and compared with mRNA expression levels. Although the ligand is expressed at highly variable amounts there is no correlation with mRNA, concordant with post-transcriptional regulation of PDGF. (0.77 MB TIF) [file pone.0007752.s007.tif]
